# Relationship with Nut Consumption for Breakfast and Postprandial Glucose, Insulin, Triglyceride Responses: A Preliminary Study from Türkiye

**DOI:** 10.3390/foods13203289

**Published:** 2024-10-17

**Authors:** Ipek Agaca Ozger, Gizem Kose, Murat Bas, Sena Oruc, Ladan Hajhamidiasl, Salim Yilmaz

**Affiliations:** 1 Department of Nutrition and Dietetics, Institute of Health Sciences, Acibadem Mehmet Ali Aydinlar University, Istanbul 34752, Türkiye; ipek.ozger@live.acibadem.edu.tr (I.A.O.); ladan.hajhamidiasl@live.acibadem.edu.tr (L.H.); 2 Department of Nutrition and Dietetics, Faculty of Health Sciences, Acibadem Mehmet Ali Aydinlar University, Istanbul 34752, Türkiye; murat.bas@acibadem.edu.tr (M.B.); sena.oruc@acibadem.edu.tr (S.O.); 3 Department of Nutrition and Dietetics, Institute of Graduate Education, Istinye University, Istanbul 34010, Türkiye; 4 Department of Health Management, Faculty of Health Sciences, Acibadem Mehmet Ali Aydinlar University, Istanbul 34752, Türkiye; salim.yilmaz@acibadem.edu.tr

**Keywords:** nuts, breakfast, taste, insulin, blood glucose, triglyceride

## Abstract

This study aimed to determine the effect of adding nuts to a regular breakfast on blood glucose, insulin, and triglyceride levels, and to evaluate participants’ opinions by sex as a preliminary study for future studies. Data and biochemical parameters were collected through face-to-face interviews with Acibadem Hospital employees (n = 12) between April and September 2023. Participants consumed 30 g of nut-supplemented breakfasts for 4 weeks while maintaining their regular lifestyle. Blood tests and visual analog scale (VAS) parameters were assessed on intervention days. VAS scores indicated differences in meal taste, post-breakfast well-being, satiety, and meal adequacy, and for “feeling better after breakfast”, and “providing better satiety”, were significantly higher for the nut-supplemented breakfasts (*p* < 0.05), especially walnut-supplemented. Under the control of age, sex, and BMI variables in the participants, women responded better to peanut butter consumption than men in terms of blood insulin and triglyceride regulation (*p* < 0.05). However, triglyceride regulation in men was better managed by walnut consumption than in women. Understanding how nut-supplemented breakfasts impact blood glucose, insulin, and triglyceride levels, as well as consumer perceptions, is crucial for promoting healthier dietary choices.

## 1. Introduction

Breakfast is still the most important meal [1] yet, not all types of breakfast are not. Breakfast requires some nutritious ingredients and quality to be the one of the key indicators of a human being’s health [2,3]. Circadian regulation of the body requires nutritious meals in the morning because the use of nutrients and energy production are better in the first morning hours [4,5]. Additionally, the circadian rhythm affects choosing food and the timing of food consumption, particularly during breakfast [6,7]. In metabolic homeostasis, nutrient metabolism regulation is arranged by hormonal processes [8], and glucose metabolism is an important driver of the metabolism of other nutrients. Insulin sensitivity and secretion are decreased between 3 a.m. and 5 a.m., and cortisol production is increased at 5 a.m. [9]. These regulators govern glucose metabolism and regulation [10]. With these regulatory procedures, a growing body of evidence has shown that the composition of breakfast shapes the food intake for the rest of the day [11,12].

Having breakfast with a good source of protein and fiber and low glycemic load is beneficial to making healthier choices during the day and provides better regulation of nutritional metabolic processes. Studies have shown that having breakfast regularly increases the intake of fiber, calcium, potassium, iron, zinc, and vitamins A, C, E, B_12_, B_6_, and B_2_ and decreases the intake of cholesterol and fats [13,14]. Nuts and seeds are found to be healthy food choices with a good source of protein, essential amino acids, minerals, vitamins, fiber, antioxidants, and polyunsaturated fatty acids; therefore, breakfasts that have nuts and seeds in them are considered as one of the healthy breakfast types [5,15,16].

In the literature, regular nut consumption is associated with improvement in appetite control, decreased energy intake, higher level of consumption of some nutrients such as fiber, plant-based protein, etc., and reduced inflammatory markers [17,18,19]. However, to provide health benefits, the timing and dosage of the consumption of nuts need to be emphasized. It is recommended to consume nuts more than five times in a week with a portion higher than 30 g each time to obtain the health benefits, except in the case of Brazil nuts (5–13 g) [20,21]. Nuts and seeds offer better postprandial glucose, insulin, and ghrelin responses with regular and sufficient consumption [22,23], yet there is lack of evidence regarding examination of the postprandial triglyceride levels after nut-supplemented meal consumption. Many studies have claimed that nut consumption in meals improves satiety and reduces hunger over time by examining appetite biomarkers in the blood [24]. However, fewer studies focus on actual food intake and people’s perceptions after consuming nut-supplemented meals.

Individuals need to be regularly encouraged to consume breakfast with healthy foods; therefore, it is required to create new alternatives by adding healthier foods to regular breakfast options and to understand their effects on human metabolism and consumer opinion. This study aimed to determine the effects of nut-supplemented breakfast on blood glucose, insulin, and triglyceride levels and on the perceptions of participants as a preliminary study for future studies.

## 2. Materials and Methods

### 2.1. Participants and Methods

This single-center nutritional intervention study was conducted between April and September 2023. For this study, an announcement about the study process was made in different working areas, and 6 women from a private dental clinic and 6 men from a private laboratory volunteered to participate in this study, for a total sample size of 12, which was convenient for power analysis results. The exclusion criteria for the study were as follows: individuals having any types of chronic diseases or dysregulation of serum glucose or insulin levels and those having food allergies or intolerances. Individuals who are sedentary, with a BMI of 20–30 kg/m^2^, and aged 20–45 years were included. All participants were verbally informed about the study, and written consent was obtained from all participants. This study was approved by the Clinical Research Ethics Committee of Acibadem University (decision number: 2022/12-22).

### 2.2. Study Conduct

**Figure 1:**
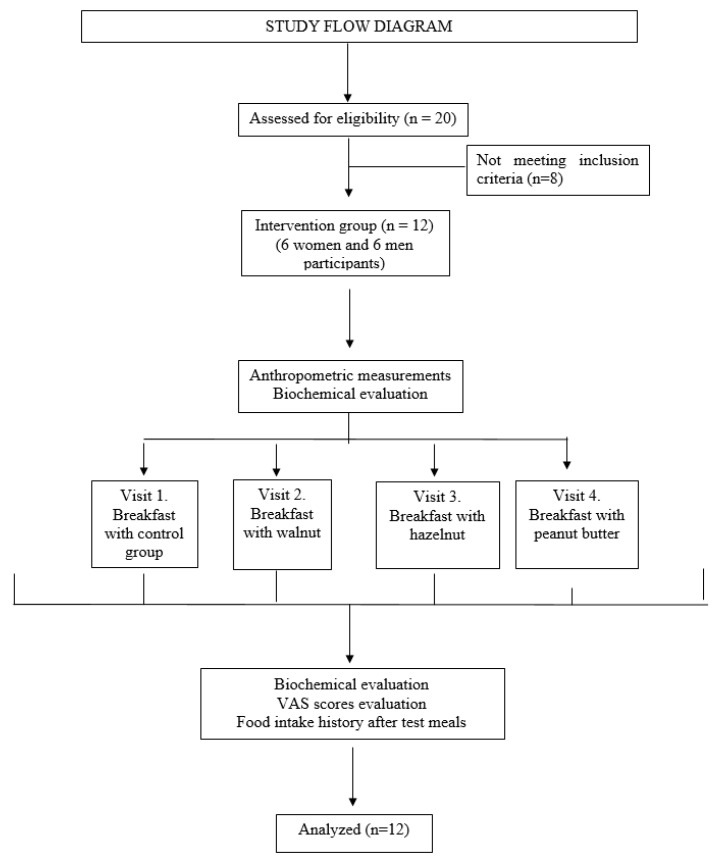
Study flow diagram.

The participants were requested to consume a test breakfast on the intervention days for 4 weeks, with no change in their nutritional routine, except on the intervention days. In this study, four test breakfasts were consumed by the participants in consecutive weeks without knowing the content of the breakfast. The participants were instructed to consume a control breakfast (Cb), a breakfast with walnut (Wb), a breakfast with hazelnut (Hb), and a breakfast with peanut butter (Pb). Unroasted and unsalted raw nuts were used in this study. Peanut butter was preferred to be sugar-free with no additives. The participants were requested to consume the test breakfasts within 15 min. It was stated that they could only drink water during breakfast; and consumption of tea and coffee was not allowed. Figure 1 shows a diagram of the study flow.

### 2.3. Test Meals and Study Planning

On the intervention days, each participant received the test breakfast that was portioned and delivered in packages and ready to eat. After consumption, the participants were instructed to record food intake during the intervention days. Furthermore, 30 g of different types of raw nuts was added to the test meals, except for the control breakfast (Cb), to observe the effect of adding nuts to the meals on some physiological processes. The Cb meal comprised boiled eggs (30 g), feta cheese (30 g), and wholegrain bread (25 g), whereas the other test meals comprised 30 g of different nuts. The contents of the test meals are presented in Table 1.

The energy and macronutrient distribution of the breakfast meal options provided to the participants is shown in Table 2.

### 2.4. Questionnaires

The survey form describing the general information, sociodemographic characteristics, and nutritional habits of the participants was completed by the researcher during a face-to-face interview. Furthermore, the participants completed the visual analog scale (VAS: 0 to 10) after each breakfast consumption, and their conditions before and after meals were analyzed. Six questions were asked to the participants in the VAS used in this study, and these questions were as follows: “How hungry were you before breakfast?” “Rate the taste of the meal you ate?” “How happy did you feel after breakfast?”, “How full did you feel after breakfast?”, and “How is your gas/bloating after breakfast?”.

### 2.5. Food Consumption Record

The participants were requested to record their food consumption on the intervention days without making any changes to their nutritional routine. They received a food record form from the researcher. Food consumption records were analyzed using the Nutrition Information System Program (BeBiS) (Ebispro for Windows, Stuttgart, Germany; Turkish version/BeBiS 7). In addition to recording the nutritional composition of foods, BeBiS can be used to create new recipes and detect the nutritional composition of the recipes. The test breakfasts were first determined in BeBiS, which then provided the nutritional composition of the test breakfasts.

### 2.6. Biochemical Parameters

The participants’ blood glucose, insulin, and triglyceride profiles (Cobas Integra 400 plus, Roche Diagnostic, Basel, Switzerland) were obtained from the values recorded during the screening interview at Acibadem LabMed, Istanbul, Turkey. For biochemical measurements, the participants were asked to fast for 12 h before the intervention days. The blood samples of the participants were provided at 0 (baseline), 60, 120, and 240 min after each breakfast consumption to obtain serum glucose, insulin, and triglyceride, under sterile conditions. The mixed meal tolerance test was not applied because it is used in diagnosing hypoglycemia, and participants with hypoglycemia were excluded from this study.

### 2.7. Anthropometric Measurements

At the beginning of the study, anthropometric measurements were made by the researcher with a bioelectrical impedance device (Tanita BC418, Tokyo, Japan) on the participants who agreed to participate in the study. After ensuring that the participants met the inclusion criteria, the measurements were repeated and if there was a difference of 1% or more between the two measurements, the measurement was repeated. The results of the three measurements were recorded on the forms by taking the average of the two closest measurements.

Body weight (kg), height (cm), body mass index (kg/m^2^), body composition (body fat mass, body fat percentage, and basal metabolic rate), and waist and hip circumference were taken. Body weight and body composition analyzes of the participants were evaluated with the Tanita BC418 model bioelectric impedance device (Tanita BC418, Tokyo, Japan), body composition analyses were applied following the standards [25]. Height was measured by using a stadiometer. The waist and hip circumference of the participants was measured and classified by following the standard [26].

### 2.8. Statistical Analysis

Descriptive statistics for categorical variables (e.g., demographic characteristics) are presented as frequencies and percentages. The conformity of numerical variables to the normal distribution was checked using the Shapiro–Wilk test. The independent sample t-test was used to compare two independent groups with normally distributed data, and the Mann–Whitney U test was used for non-normally distributed data. In analyses involving covariance analysis (ANCOVA) and repeated measures, repeated measures ANCOVA was used to evaluate estimated marginal means and differences between groups. Data analysis was performed using SPSS 25.0 and R 4.4.1 software.

## 3. Results

In Table 3, a comparison of various demographic characteristics and anthropometric measurements between men and women in this study sample of 12 participants (6 men and 6 women) were shown. The measures include age, body mass index (BMI), waist circumference, hip circumference, waist-to-height ratio, waist-to-hip ratio, body fat mass, body fat percentage, and basal metabolic rate (BMR). Men generally had significantly higher BMI, waist circumference, hip circumference, waist-to-height ratio, waist-to-hip ratio, body fat mass, and BMR compared to women, as expected. No significant differences were observed in age and body fat percentage between the sexes.

The VAS scores for hunger before breakfast showed no significant differences among the walnut (Wb), hazelnut (Hb), peanut butter (Pb), and control (Cb) breakfasts (F = 0.348, *p* > 0.05). Similarly, no significant differences were observed among women regarding hunger before breakfast (F = 1.112, *p* = 0.369). There were also no significant differences among men for hunger scores between the groups (F = 0.198, *p* = 0.897) (Table 4).

The VAS scores for taste of meal varied significantly among different breakfast types (F = 10.263, *p* < 0.001). Specifically, the walnut breakfast (Wb) had a significantly higher score compared to hazelnut (Hb) and control (Cb) breakfasts. Among women, the taste of walnut breakfast (Wb) was rated significantly higher than that of peanut butter (Pb), control (Cb), and hazelnut (Hb) breakfasts (F = 9.812, *p* < 0.001). Similarly, among men, the walnut breakfast (Wb) had a significantly higher score compared to peanut butter (Pb), hazelnut (Hb), and control (Cb) breakfasts (F = 4.155, *p* = 0.020) (Table 4).

The scores for feeling good after breakfast differed significantly according to breakfast type (F = 24.913, *p* < 0.001). The walnut (Wb) and peanut butter (Pb) breakfasts were rated higher than the hazelnut (Hb) and control (Cb) breakfasts. Among women, the walnut breakfast (Wb) had a significantly higher score compared to all other breakfasts (Hb, Pb, Cb) (F = 26.185, *p* < 0.001). Similarly, among men, the walnut breakfast (Wb) was rated higher compared to the hazelnut and peanut butter breakfasts (F = 7.030, *p* = 0.002) (Table 4).

The satiety after breakfast scores showed significant differences among breakfast types (F = 16.320, *p* < 0.001). The walnut (Wb) and peanut butter (Pb) breakfasts provided a higher satiety feeling compared to the hazelnut (Hb) and control (Cb) breakfasts. Among women, the walnut breakfast (Wb) created a higher satiety feeling compared to other breakfasts (Hb, Pb, Cb) (F = 13.486, *p* < 0.001). Similarly, among men, the walnut breakfast (Wb) was rated higher than the hazelnut and peanut butter breakfasts (F = 7.030, *p* = 0.002) (Table 4).

The scores for the adequacy of breakfast showed significant differences among breakfast types (F = 8.521, *p* < 0.001). The walnut breakfast (Wb) was rated higher compared to the hazelnut (Hb), peanut (Pb) and control (Cb) breakfasts. Among women, the walnut breakfast (Wb) was rated significantly higher compared to Hb and Cb breakfasts (F = 12.178, *p* = 0.014). Among men, the walnut breakfast (Wb) was also rated higher compared to the hazelnut and control breakfasts (F = 5.481, *p* = 0.006) (Table 4).

The participants’ daily total energy and macronutrient intakes (g and % of total energy) were compared according to breakfast with different types of nuts, as shown in Table 5. The contents of CHO (%), protein (g), protein (%) and fat (%) were not significantly different between nut-supplemented breakfasts. The mean total energy intake (kcal) in Cb (1539.43 ± 319.11) was higher than that in Pb (1115.58 ± 372.56) and Wb (1058.70 ± 319.96) (F = 5.751; *p* < 0.05). These energy differences were related to carbohydrate (CHO) and fat intake. The mean CHO (g) intake in Cb (153.80 ± 43.26) was higher than that in Pb (101.12 ± 36.61) (F = 3.376; *p* < 0.05), and the mean fat (g) intake in Cb (70.98 ± 18.44) was higher than that in Pb (49.39 ± 21.99) and Wb (46.30 ± 11.54) (F = 5.584; *p* < 0.05) (Table 5).

When comparing the changes in biochemical parameters (fasting blood glucose, 60th minute blood glucose, 120th minute, and 240th minute blood glucose) between the walnut, hazelnut, peanut butter, and control groups, under the control of age, sex, and BMI variables, no statistically significant differences were found (F: 2.368; *p* = 0.085). However, at the 90% confidence level, the control group was found to be statistically partially significantly higher compared to the hazelnut group (estimated marginal mean difference: 9.604; *p* = 0.093). Under the same control variables, no statistically significant differences were found in the changes in insulin measurements between the groups (F: 1.374; *p* = 0.264). A similar situation applies to triglyceride (F: 0.633; *p* = 0.598) (Table 6 and Table S1). When compared by sex, the difference in Wb–triglyceride values were higher in women than in men. Men had higher Pb–insulin and Pb–triglyceride difference values than women (*p* < 0.05). Regarding Cb–blood glucose difference values, women had a higher increase than men (*p* < 0.001) (Table 6 and Table S1).

## 4. Discussion

In this study, four test breakfasts were prepared by the researchers and were named as control breakfast (Cb), breakfast with walnut (Wb), breakfast with hazelnut (Hb), and breakfast with peanut butter (Pb). This study primarily aimed to determine the effects of nuts on the regular breakfast model (Cb) on blood glucose, insulin, and triglyceride levels and to evaluate participants’ opinions about breakfasts via VAS scoring. Finally, this study revealed differences between the sexes.

The pleasantness of a meal and the composition of the meal affect food intake later on [27,28]. Furthermore, the different types of carbohydrates (high-fiber and low-fiber content) and fat content of meals given to individuals change their satiety and post-meal alertness [28]. Because pleasantness is important for the meal and can shape the rest of food consumption, it is important to arrange nutritious additions to breakfast that can help control nutritional behavior and food intake. In this study, different types of nuts were added to the regular type of breakfast that contains egg, feta cheese, and wholegrain bread. According to the VAS scores provided by the participants for each test breakfast, the participants found that nut-supplemented breakfasts (i.e., Wb, Hb, and Pb) “tasted better”, “felt better after breakfast”, “provided better satiety”, and “had better adequacy”. Furthermore, Wb had the highest VAS score in each category. Participants had significantly lower energy intake throughout the day when they consumed nut-supplemented breakfasts compared to the control breakfast. Additionally, the VAS scores for “felt better after breakfast”, and “provided better satiety”, were significantly higher for the nut-supplemented breakfasts. This study’s conclusion is promising for controlling food intake with additional nut consumption in the first meal of the day, but further and longer-term interventions are needed to fully understand the actual impacts, as there is a lack of evidence in this area.

Individuals should be encouraged to consume breakfast rich in protein, carbohydrates, and unsaturated fatty acids, because these nutrients are beneficial to regulate metabolic processes according to the circadian rhythm [29,30]. In this study, the participants received 5 g of protein, 6 g of carbohydrates, and 27 g of fat from Wb, Hb, and Pb and had increased energy content by 95% compared with that of Cb. Even though the participants almost duplicated their energy intake with the nut-supplemented breakfasts, Cb resulted in significantly higher energy intake than Wb and Pb throughout the intervention days. The literature recommends the consumption of ≥5 servings per week of nuts because consumption of nuts improves blood glucose regulation, insulin sensitivity [31], appetite, and food consumption [32] and decreases inflammation [33]. In this study, adding one serving of different types of nuts to the breakfasts resulted in significantly better regulation of blood glucose and insulin levels in all participants. Further studies may examine inflammatory criteria.

The literature presents conflicting findings on the impact of nut consumption on blood glucose levels, but it is evident that nut consumption shows a stronger effect within the first two hours after eating [24,34]. Our study’s findings align with the literature in this regard. In the study, different fluctuations in blood glucose levels were observed from 0 to 240 min across all groups, with no significant differences. Under the control of age, sex, and BMI variables, women responded better to peanut butter consumption than men in terms of blood insulin and triglyceride regulation. However, triglyceride regulation in men was better managed by walnut consumption than in women. According to the literature, there is no significant impact of nut consumption on lipid profiles; it can vary depending on the type of the nut [35]. While previous research has shown that pistachio [36] and almond consumption [37] are more effective in improving triglyceride levels, our findings revealed that a walnut-supplemented meal (Wb) resulted in significantly better control of triglyceride levels in men and women, mostly men. Given the lack of evidence on the short-term impact of nut consumption, this promising effect of walnuts warrants further investigation but remains open to discussion. The data gathered in this study align with the existing literature on the effects of nut consumption and the assumptions made in relevant studies [24,34]. However, there is a notable lack of evidence specifically addressing the impact of nut consumption at breakfast on food intake and metabolic processes. Therefore, this study offers promising insights that could guide future intervention research on nut consumption.

### Strengths and Limitations

This study was planned as a preliminary study for future research with a few limitations that should be emphasized. Although the sample size was determined via power analysis, larger sample sizes are suggested for future studies. The heterogeneity of the participants is well known but the statistical analysis was applied by consideration. As the study was designed as a one-meal intervention, other meal consumptions may have affected the blood parameters. However, compared with the baseline, the results are still valuable.

## 5. Conclusions

In conclusion, this study underscores the importance of incorporating nutritious foods like nuts into breakfast to enhance metabolic health and overall well-being. Encouraging regular consumption of healthy breakfasts with components such as nuts (especially walnut and hazelnut) can lead to better dietary choices throughout the day, promoting long-term health benefits and enhancing meal satisfaction and satiety.

## Figures and Tables

**Table 1:** Contents of test meals (g).

	Control Breakfast (Cb)	Breakfast with Walnut (Wb)	Breakfast with Hazelnut (Hb)	Breakfast with Peanut Butter (Pb)
Egg	30	30	30	30
Feta cheese	30	30	30	30
Wholegrain bread	25	25	25	25
Walnut	-	30	-	-
Hazelnut	-	-	30	-
Peanut butter	-	-	-	30

**Table 2:** Energy and macronutrient contents of the test breakfast meals.

	CHO (g)	Protein (g)	Fat (g)	Energy (kcal)
Test breakfast 0				
1 egg (30 g)	0.6	6.3	5.3	77.6
Feta cheese (30 g)	1.2	4.8	6.5	82.6
Wholegrain bread (25 g)	10.2	1.9	0.2	50.9
Nuts				
Walnut	4.1	4.6	19.6	196.2
Hazelnut	5.0	4.5	18.2	188.4
Peanut butter	6.3	7.3	14.9	176.1
Test breakfasts 1–4				
Breakfast with control group (Cb)	12	13	12	211.1
Breakfast with walnut (Wb)	16.1	17.6	31.6	407.3
Breakfast with hazelnut (Hb)	17	17.5	30.2	398.9
Breakfast with peanut butter (Pb)	18.3	20.3	26.9	387.2

**Table 3:** Demographic characteristics and anthropometric measures according to sex.

	Total (n = 12)	Men (n = 6)	Women (n = 6)	*p*-Value
	$\bar{X}\;\pm \;SD$	$\bar{X}\;\pm \;SD$	$\bar{X}\;\pm \;SD$	
Age (year)	27.75 ± 6.34	29.67 ± 7.69	25.83 ± 4.54	0.317
Body mass index (kg/m^2^)	24.16 ± 3.95	26.89 ± 2.90	21.43 ± 2.83	0.008 *
Waist circumference	84.92 ± 12.19	94.83 ± 5.42	75.00 ± 7.85	0.000 **
Hip circumference (cm)	98.83 ± 4.80	102.29 ± 2.64	95.36 ± 3.87	0.005 *
Waist/height ratio	0.49 ± 0.06	0.53 ± 0.03	0.45 ± 0.04	0.004 *
Waist/hip ratio	0.86 ± 0.09	0.93 ± 0.04	0.79 ± 0.06	0.000 **
Body fat mass (kg)	16.92 ± 5.03	19.68 ± 4.67	14.17 ± 3.94	0.052
Body fat (%)	22.71 ± 3.48	21.86 ± 1.80	23.56 ± 4.65	0.433
Basal metabolic rate	1630.82 ± 299.3	1884.81 ± 73.72	1376.83 ± 109.7	0.000 **

t: Independent t-test; U: Mann–Whitney U-test. * *p* < 0.05; ** *p* < 0.001.

**Table 4:** Modified visual analog scale parameters related to breakfast (n = 12).

	Total (n = 12)			Women (n = 6)			Men (n = 6)		
	Mean ± SD	F	*p*-Value	Mean ± SD	F_w_	*p*-Value	Mean ± SD	F_m_	*p*-Value
Hunger before breakfast									
Wb _a_	7.58 ± 1.31	4.516	0.348	7.17 ± 0.75	1.112	0.369	8.00 ± 1.67	0.198	0.897
Hb _b_	7.58 ± 1.08			7.17 ± 0.75			8.00 ± 1.26		
Pb _c_	7.08 ± 1.51			6.50 ± 1.38			7.67 ± 1.51		
Cb _d_	6.83 ± 1.40			6.17 ± 1.47			7.50 ± 1.05		
Taste of meal									
Wb _a_	7.08 ± 0.90 _>b,d_	10.263	<0.001 ***	7.67 ± 0.82 _>b_	9.812	<0.001 ***	6.50 ± 0.55 _>d_	4.155	0.020 *
Hb _b_	5.00 ± 0.74			4.67 ± 0.52			5.33 ± 0.82		
Pb _c_	6.17 ± 1.03 _>b_			6.17 ± 1.47 _>b_			5.67 ± 1.03		
Cb _d_	5.50 ± 1.45			6.67 ± 0.82			4.83 ± 1.17		
Feeling good after breakfast									
Wb _a_	7.92 ± 1.24 _>b,d_	24.913	<0.001 ***	9.00 ± 0.63 _>b,c,d_	26.185	<0.001 ***	6.83 ± 0.41 _>d_	7.030	0.002 **
Hb _b_	5.67 ± 0.78			5.67 ± 0.82			5.67 ± 0.82		
Pb _c_	6.92 ± 1.38 _>b,d_			7.50 ± 1.38 _>b,d_			6.33 ± 1.21 _>d_		
Cb _d_	4.75 ± 0.45			4.67 ± 0.52			4.83 ± 0.41		
Satiety after breakfast									
Wb _a_	8.25 ± 0.97 _>b,c,d_	16.320	<0.001 ***	8.83 ± 0.75 _>b,d_	13.486	<0.001 ***	7.67 ± 0.82 _>b,c,d_	6.235	0.003 **
Hb _b_	5.67 ± 1.07			6.00 ± 0.89			5.33 ± 1.21		
Pb _c_	6.58 ± 1.68 _>d_			7.50 ± 0.84 _>d_			5.67 ± 1.86		
Cb _d_	5.17 ± 1.47			5.83 ± 1.17			4.50 ± 1.52		
Adequacy of breakfast									
Wb _a_	8.42 ± 1.16 _>b,c,d_	8.521	<0.001 ***	9.00 ± 0.63 _>b,d_	12.178	0.014 *	7.83 ± 1.33 _>b,c_	5.481	0.006 **
Hb _b_	6.42 ± 1.38			7.50 ± 0.55			5.33 ± 1.03		
Pb _c_	7.17 ± 1.70			7.50 ± 1.38			5.83 ± 1.33		
Cb _d_	6.75 ± 1.36			8.50 ± 0.55			6.00 ± 0.89		

F: ANCOVA and covariates: age, sex and BMI; F_w_: ANCOVA and covariates: age and BMI among women; F_m_: ANCOVA and covariates: age and BMI among men; control breakfast (Cb), breakfast with walnut (Wb), breakfast with hazelnut (Hb), and breakfast with peanut butter (Pb); a: Wb, b: Hb, c: Pb, d: Cb; * *p* < 0.05; ** *p* < 0.01; ***: *p* < 0.001.

**Table 5:** Total daily energy and macronutrient intakes according to sex according to breakfast with different types of nuts.

	Total Energy	CHO (g)	CHO (%)	Protein (g)	Protein (%)	Fat (g)	Fat (%)
Wb _a_	1.058.70 ± 319.96	110.18 ± 44.12	42.00 ± 8.74	45.51 ± 19.50	17.00 ± 3.38	46.30 ± 11.54	39.01 ± 7.32
Hb _b_	1.204.96 ± 329.69	126.16 ± 55.40	41.60 ± 10.18	42.21 ± 16.77	14.40 ± 3.77	53.02 ± 13.31	39.80 ± 5.62
Pb _c_	1.115.58 ± 372.56	101.12 ± 36.61	41.32 ± 7.62	47.58 ± 16.86	17.73 ± 3.97	49.39 ± 21.99	39.25 ± 9.24
Cb _d_	1539.43 ± 319.11 _>a,c_	153.80 ± 43.26 _>c_	41.42 ± 9.48	59.02 ± 22.52	15.67 ± 4.12	70.98 ± 18.44 _>a,b,c_	40.83 ± 5.13
F	5.751	3.376	0.013	1.829	1.788	5.584	0.155
*p*	0.002 **	0.027 *	*0.998*	*0.156*	*0.164*	0.003 **	*0.926*

F: ANCOVA and covariates: age, sex and BMI; control breakfast (Cb), breakfast with walnut (Wb), breakfast with hazelnut (Hb), and breakfast with peanut butter (Pb); statistical significance: a > b > c, no letter means no statistical significance, * *p* < 0.05; ** *p* < 0.001.

**Table 6:** Differences in changes in blood glucose and insulin parameters with consumption of breakfast with different types of nuts.

	EM Means	Standard Error	%95 LB	%95 UB	F	*p*	Post-hoc
Wb–Blood Glucose	95.958	2.688	90.529	101.387	2.368	0.085	Hb < Cb *_(%10 margin of error)_*
Hb–Blood Glucose	88.438	2.688	83.009	93.866			
Pb–Blood Glucose	93.521	2.688	88.092	98.950			
Cb–Blood Glucose	98.042	2.688	92.613	103.471			
Wb–Blood Insulin	11.469	1.304	8.834	14.103	1.374	0.264	-
Hb–Blood Insulin	11.315	1.304	8.681	13.949			
Pb–Blood Insulin	13.136	1.304	10.502	15.770			
Cb–Blood Insulin	14.549	1.304	11.915	17.183			
Wb–Triglyceride	129.812	11.072	107.453	152.172	0.633	0.598	-
Hb–Triglyceride	141.271	11.072	118.911	163.630			
Pb–Triglyceride	120.021	11.072	97.661	142.380			
Cb–Triglyceride	127.396	11.072	105.036	149.755			

F: repeated measures ANCOVA and covariates: age, sex, BMI; time Points: 0 min, 60 min, 120 min, 240 min; control breakfast (Cb), breakfast with walnut (Wb), breakfast with hazelnut (Hb), and breakfast with peanut butter (Pb).

## Data Availability

The original contributions presented in the study are included in the article and Supplementary Materials, further inquiries can be directed to the corresponding author.

## Supplementary Materials

The following supporting information can be downloaded at: https://www.mdpi.com/article/10.3390/foods13203289/s1, Table S1: Comparison of blood glucose, insulin, and triglyceride parameters (baseline, 60 min, 120 min, and 240 min) with consumption of breakfast with different kinds of nuts according to sex.
