# Supplementary material for: Relationship with Nut Consumption for Breakfast and Postprandial Glucose, Insulin, Triglyceride Responses: A Preliminary Study from Türkiye
Source: Foods. 2024 Oct 17;13(20):3289. doi: 10.3390/foods13203289 (PMC11508106; doi:10.3390/foods13203289)
Supplement: Supplementary file 1 [file foods-13-03289-s001.zip › foods-3214080-supplementary.pdf]

**Table S1.** Comparison of blood glucose, insulin, and triglyceride parameters (baseline, 60 min, 120 min, and 240 min) with consumption of breakfast with different kinds of nuts according to sex.

| | Total (n = 12) | | | | | | | |
|---|---|---|---|---|---|---|---|---|
| | 0 min | | 60 min | | 120 min | | 240 min | |
| BG | $\bar{X} \pm SS$ | Median (min–max) | $\bar{X} \pm SS$ | Median (min–max) | $\bar{X} \pm SS$ | Median (min–max) | $\bar{X} \pm SS$ | Median (min–max) |
| Wb–BG | 92.25 ± 11.25 | 91 (72–112) | 105.33 ± 10.02$^{ab}$ | 105.5 (90–120) | 95.50 ± 6.02 | 94 (85–105) | 90.75 ± 5.86$^{b}$ | 90 (78–100) |
| Hb–BG | 89.33 ± 8.33 | 90 (73–104) | 97.17 ± 14.36$^{a}$ | 92 (76–121) | 89.50 ± 15.38 | 89.5 (65–113) | 77.75 ± 9.94$^{a}$ | 77.5 (61–92) |
| Pb–BG | 87.50 ± 6.04 | 90 (76–96) | 117.58 ± 15.76$^{bc}$ | 120.5 (97–148) | 94.33 ± 11.72 | 91.5 (81–127) | 74.67 ± 6.47$^{a}$ | 71.5 (67–90) |
| Cb–BG | 89.42 ± 13.97 | 89.5 (72–123) | 125.42 ± 14.76$^{c}$ | 127 (104–155) | 101.58 ± 17.31 | 92 (81–128) | 75.75 ± 10.81$^{a}$ | 75.5 (65–100) |
| F-H | F = 0.432 | | F = 9.816 | | 4.077 | | F = 9.149 | |
| p | 0.731 | | <0.001** | | 0.253 | | <0.001** | |
| BI | | | | | | | | |
| Wb–BI | 5.17 ± 2.35 | 4.8 (2.6–8.8) | 24.74 ± 8.10 | 24.2 (15.4–39.3) | 10.51 ± 1.98 | 10.4 (8.1–15.2) | 5.45 ± 2.37 | 5.1$^{ab}$ (1.5–8.8) |
| Hb –BI | 6.91 ± 4.18 | 6.7 (1.2–13) | 25.29 ± 10.91 | 25.9 (7–42.2) | 9.53 ± 3.79 | 10.3 (2.9–14.3) | 3.53 ± 2.15 | 2.4$^{a}$ (2–8.3) |
| Pb –BI | 8.14 ± 6.10 | 5.7 (0.9–19.7) | 24.67 ± 12.15 | 28.1 (6.8–48.1) | 13.13 ± 6.82 | 11.4 (3.7–30.6) | 6.60 ± 3.37 | 5.9$^{b}$ (2.9–15) |
| Cb–BI | 8.21 ± 4.99 | 7.1 (2.7–17) | 32.19 ± 15.89 | 30.8 (11.2–76) | 12.57 ± 6.26 | 10.9 (4.1–23.7) | 5.22 ± 4.96 | 3.7$^{ab}$ (1.2–18.8) |
| F-H | F = 1.142 | | 2.398 | | F = 1.334 | | 8.379 | |
| p | 0.342 | | 0.494 | | 0.276 | | 0.039* | |
| TG | | | | | | | | |
| Wb–TG | 133.67 ± 115.59 | 95.5 (39–382) | 141.42 ± 116.43 | 109.5 (31–389) | 150.67 ± 114.24 | 118.5 (30–391) | 93.50 ± 29.57 | 102 (49–152) |
| Hb –TG | 122.50 ± 88.55 | 83.5 (62–356) | 130.25 ± 91.15 | 88 (68–367) | 140.75 ± 98.44 | 93 (67–385) | 171.58 ± 119.33 | 117.5 (70–410) |
| Pb –TG | 104.92 ± 72.48 | 75.5 (48–275) | 118.92 ± 71.17 | 88.5 (64–280) | 126.58 ± 71.94 | 96 (77–290) | 129.67 ± 82.34 | 109 (62–305) |
| Cb–TG | 128.33 ± 102.09 | 85 (30–350) | 122.42 ± 91.58 | 97.5 (31–355) | 129.17 ± 91.30 | 91.5 (27–356) | 129.67 ± 95.68 | 94.5 (27–360) |
| H | 0.360 | | 0.230 | | 0.328 | | 3.247 | |
| p | 0.948 | | 0.973 | | 0.955 | | 0.355 | |
| | Men (n = 6) | | | | | | | |
| | 0 min | | 60 min | | 120 min | | 240 min | |
| BG | $\bar{X} \pm SS$ | Median (min–max) | $\bar{X} \pm SS$ | Median (min–max) | $\bar{X} \pm SS$ | Median (min–max) | $\bar{X} \pm SS$ | Median (min–max) |
| Wb–BG | 99.67 ± 8.12 | 100.5$^{b}$ (90–112) | 104.00 ± 9.59 | 104 (92–115) | 97.83 ± 5.60 | 98 (91–105) | 93.83 ± 4.67 | 93.5$^{c}$ (88–100) |
| Hb–BG | 91.67 ± 5.01 | 90.5$^{ab}$ (86–101) | 101.67 ± 12.42 | 98 (90–118) | 93.00 ± 17.33 | 90.5 (65–113) | 82.17 ± 7.57 | 80$^{b}$ (75–92) |
| Pb–BG | 87.00 ± 6.93 | 87.5$^{a}$ (76–96) | 111.33 ± 15.38 | 107.5 (97–130) | 90.00 ± 5.66 | 91 (81–98) | 71.00 ± 2.61 | 71$^{a}$ (67–75) |
| Cb–BG | 99.33 ± 12.24 | 95.5$^{ab}$ (90–123) | 117.67 ± 13.52 | 114 (104–136) | 94.17 ± 16.14 | 89.5 (81–126) | 73.00 ± 13.9 | 76$^{ab}$ (65–100) |
| F-H | 8.617 | | F = 1.910 | | 3.862 | | 12.646 | |
| p | 0.035* | | 0.161 | | 0.277 | | 0.005** | |
| BI | | | | | | | | |
| Wb–BI | 4.48 ± 2.20 | 3.7 (2.6–8.3) | 22.17 ± 8.79 | 18.1 (16.6–39.3) | 10.40 ± 2.68 | 9.9 (8.1–15.2) | 5.57 ± 1.46 | 5.1$^{b}$ (4.3–8.4) |
| Hb–BI | 6.10 ± 4.71 | 4.6 (1.2–13) | 24.18 ± 13.46 | 24 (7–42.2) | 7.58 ± 4.00 | 7.3 (2.9–13) | 2.97 ± 1.85 | 2.2$^{a}$ (2–6.7) |
| Pb–BI | 4.65 ± 3.90 | 3.9 (0.9–12) | 16.95 ± 9.63 | 15.8 (6.8–29) | 10.05 ± 4.58 | 9.5 (3.7–16) | 5.27 ± 1.98 | 5$^{ab}$ (2.9–7.8) |
| Cb–BI | 9.42 ± 6.05 | 9.4 (2.7–17) | 24.30 ± 8.68 | 24 (11.2–34) | 10.57 ± 7.01 | 8.2 (4.1–23.7) | 4.10 ± 2.31 | 3.9$^{ab}$ (1.5–8.2) |
| F-H | F = 1.591 | | 2.124 | | F = 0.502 | | 7.925 | |
| p | 0.223 | | 0.547 | | 0.685 | | 0.048* | |
| TG | | | | | | | | |
| Wb–TG | 197.17 ± 137.05 | 145 (65–382) | 202.00 ± 139.21 | 147.5 (70–389) | 209.00 ± 136.38 | 156.5 (86–391) | 101.33 ± 13.68 | 107.5 (75–110) |
| Hb–TG | 164.00 ± 109.99 | 124.5 (67–356) | 168.83 ± 113.14 | 127.5 (69–367) | 180.83 ± 119.94 | 132 (76–385) | 203.33 ± 123.56 | 153.5 (98–410) |

| | | | | | | | | |
|---|---|---|---|---|---|---|---|---|
| Pb–TG | 144.50 ± 87.37 | 109 (65–275) | 145.17 ± 89.22 | 103 (70–280) | 158.17 ± 90.10 | 110 (86–290) | 180.83 ± 89.07 | 141 (99–305) |
| Cb–TG | 185.67 ± 116.90 | 153 (64–350) | 172.67 ± 103.62 | 156.5 (67–355) | 175.33 ± 101.77 | 160 (76–356) | 178.67 ± 104.02 | 162.5 (72–360) |
| F-H | F = 0.251 | | F = 0.256 | | 0.364 | | 5.660 | |
| p | 0.860 | | 0.856 | | 0.948 | | 0.129 | |
| Women (n = 6) | | | | | | | | |
| | 0 min | | 60 min | | 120 min | | 240 min | |
| BG | $\bar{X} \pm SS$ | Median (min–max) | $\bar{X} \pm SS$ | Median (min–max) | $\bar{X} \pm SS$ | Median (min–max) | $\bar{X} \pm SS$ | Median (min–max) |
| Wb–BG | 84.83 ± 8.98 | 84.5 (72–99) | 106.67 ± 11.17$^{ab}$ | 105.5 (90–120) | 93.17 ± 5.95$^{ab}$ | 92 (85–101) | 87.67 ± 5.57$^{b}$ | 88 (78–95) |
| Hb–BG | 87.00 ± 10.70 | 84.5 (73–104) | 92.67 ± 15.83$^{a}$ | 88.5 (76–121) | 86.00 ± 13.80$^{a}$ | 82.5 (74–111) | 73.33 ± 10.63$^{a}$ | 72 (61–90) |
| Pb–BG | 88.00 ± 5.62 | 90 (79–95) | 123.83 ± 14.69$^{bc}$ | 125 (107–148) | 98.67 ± 15.00$^{ab}$ | 95.5 (86–127) | 78.33 ± 7.28$^{ab}$ | 79.5 (70–90) |
| Cb–BG | 79.50 ± 6.60 | 80.5 (72–89) | 133.17 ± 12.35$^{c}$ | 130 (121–155) | 109.00 ± 16.32$^{b}$ | 112.5 (88–128) | 78.50 ± 6.75$^{ab}$ | 78 (70–90) |
| F | F = 1.279 | | F = 10.466 | | F = 3.147 | | F = 3.530 | |
| p | 0.309 | | <0.001** | | 0.048* | | 0.034* | |
| BI | | | | | | | | |
| Wb–BI | 5.86 ± 2.49 | 6.2 (2.7–8.8) | 27.32 ± 7.15 | 28.1 (15.4–35.2) | 10.62 ± 1.18 | 10.4 (9.4–12.6) | 5.34 ± 3.19 | 5.4 (1.5–8.8) |
| Hb–BI | 7.72 ± 3.84 | 8.4 (2.1–11.8) | 26.40 ± 8.83 | 26.6 (14.4–39.2) | 11.47 ± 2.55 | 12.1 (7.1–14.3) | 4.10 ± 2.45 | 3.6 (2–8.3) |
| Pb–BI | 11.63 ± 6.11 | 12.2 (3.4–19.7) | 32.40 ± 9.43 | 31 (19.6–48.1) | 16.21 ± 7.65 | 13.3 (10.3–30.6) | 7.93 ± 4.11 | 7.2 (3.2–15) |
| Cb–BI | 7.01 ± 3.83 | 7.1 (2.7–13.56) | 40.08 ± 18.19 | 34 (26.9–76) | 14.57 ± 5.23 | 13.5 (10–23.7) | 6.34 ± 6.77 | 3.2 (1.2–18.8) |
| F-H | F = 2.063 | | 3.847 | | F = 1.758 | | F = 0.799 | |
| p | 0.137 | | 0.279 | | 0.188 | | 0.509 | |
| TG | | | | | | | | |
| Wb–TG | 70.17 ± 30.58 | 59 (39–115) | 80.83 ± 40.46 | 67 (31–134) | 92.33 ± 44.09 | 85 (30–144) | 85.67 ± 39.87 | 69 (49–152) |
| Hb–TG | 81.00 ± 31.91 | 68.5 (62–145) | 91.67 ± 43.65 | 75 (68–180) | 100.67 ± 55.46 | 81 (67–213) | 139.83 ± 116.8 | 92 (70–376) |
| Pb–TG | 65.33 ± 12.77 | 66 (48–84) | 92.67 ± 39.11 | 76.5 (64–167) | 95.00 ± 29.56 | 82 (77–154) | 78.50 ± 26.46 | 69 (62–131) |
| Cb–TG | 71.00 ± 37.06 | 65 (30–137) | 72.17 ± 40.66 | 61 (31–136) | 83.00 ± 53.52 | 76 (27–180) | 80.67 ± 59.65 | 62.5 (27–193) |
| H | 1.098 | | 1.958 | | 0.772 | | 3.453 | |
| p | 0.778 | | 0.581 | | 0.856 | | 0.327 | |

BG, blood glucose; BI, blood insulin; TG, triglyceride; control breakfast (Cb), breakfast with walnut (Wb), breakfast with hazelnut (Hb), and breakfast with peanut butter (Pb);
F: one-way ANOVA; H: Kruskal–Wallis H test
*$p$ < 0.05; **$p$ < 0.001
a > b > c
